# (*Z*)-4-Bromo-*N*-{(*Z*)-3-[(4-bromo-2,6-diisopropyl­phen­yl)imino]­butan-2-yl­idene}-2,6-diisopropyl­aniline

**DOI:** 10.1107/S160053681205194X

**Published:** 2013-01-09

**Authors:** Cun Zhang, Guo-Fan Wu, Bao-Mei Huang, Xiao-Quan Lu

**Affiliations:** a Key Laboratory of Bioelectrochemistry & Environmental Analysis of Gansu Province, College of Chemistry & Chemical Engineering, Northwest Normal University, Lanzhou 730070, People’s Republic of China

## Abstract

The title compound, C_28_H_38_Br_2_N_2_, is centrosymmetric with the mid-point of the central C—C bond of the butyl group located on an inversion center. The terminal benzene ring is approximately perpendicular to the central 1,4-diaza­butadiene mean plane [dihedral angle = 78.23 (3)°]. No hydrogen bonding or aromatic stacking is observed in the crystal structure.

## Related literature

For applications of diimine catalysts, see: Cotts *et al.* (2000 ▶); Ittel *et al.* (2000 ▶); Johnson *et al.* (1995 ▶); Zhang & Ye (2012 ▶).
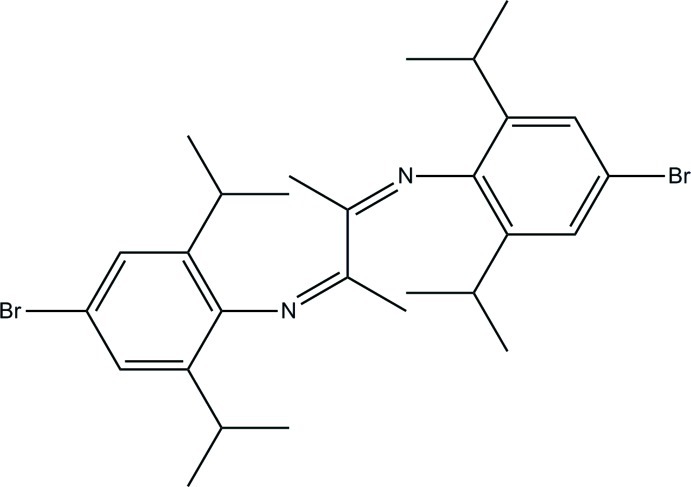


## Experimental

### 

#### Crystal data

C_28_H_38_Br_2_N_2_
*M* *_r_* = 562.42
Monoclinic, 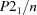

*a* = 9.099 (3) Å
*b* = 12.199 (4) Å
*c* = 13.566 (5) Å
β = 104.905 (5)°
*V* = 1455.2 (9) Å^3^
*Z* = 2
Mo *K*α radiation
μ = 2.80 mm^−1^
*T* = 296 K
0.25 × 0.23 × 0.19 mm

#### Data collection

Bruker APEXII CCD diffractometer
Absorption correction: multi-scan (*SADABS*; Bruker, 2001 ▶) *T*_min_ = 0.541, *T*_max_ = 0.618
7266 measured reflections
2685 independent reflections
1460 reflections with *I* > 2σ(*I*)
*R*_int_ = 0.043

#### Refinement

*R*[*F* ^2^ > 2σ(*F* ^2^)] = 0.052
*wR*(*F* ^2^) = 0.179
*S* = 0.94
2685 reflections
150 parameters
84 restraints
H-atom parameters constrained
Δρ_max_ = 0.46 e Å^−3^
Δρ_min_ = −0.40 e Å^−3^

### 

Data collection: *APEX2* (Bruker, 2007 ▶); cell refinement: *SAINT* (Bruker, 2007 ▶); data reduction: *SAINT*; program(s) used to solve structure: *SHELXTL* (Sheldrick, 2008 ▶); program(s) used to refine structure: *SHELXTL*; molecular graphics: *SHELXTL*; software used to prepare material for publication: *SHELXTL*.

## Supplementary Material

Crystal structure: contains datablock(s) I, global. DOI: 10.1107/S160053681205194X/xu5664sup1.cif

Structure factors: contains datablock(s) I. DOI: 10.1107/S160053681205194X/xu5664Isup2.hkl

Supplementary material file. DOI: 10.1107/S160053681205194X/xu5664Isup3.cml

Additional supplementary materials:  crystallographic information; 3D view; checkCIF report

## Figures and Tables

**Fig. 1.**
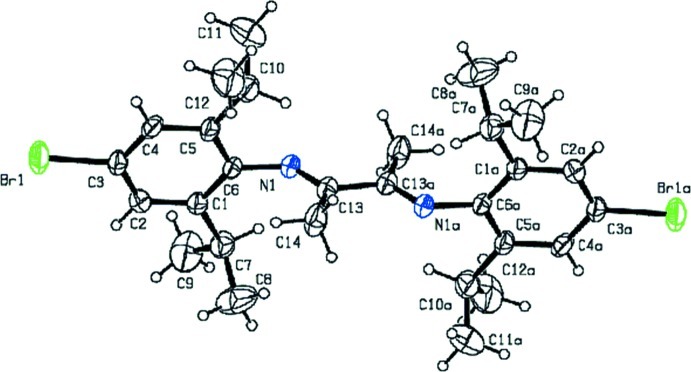
Molecular structure of the title compound, using 30% probability level ellipsoids.

## supplementary crystallographic information

### Comment

In recent years, Ni(II)/Pd(II)-α-diimine catalysts were greatly attracted attention due to their high catalytic activity and influence on product performance in olefin polymerization (Zhang & Ye, 2012; Johnson *et al.*, 1995). It is well known that the polymerization conditions (such as the reaction olefin pressure, temperature *etc.*) and ligand structure had a great impact on catalytic activity and polymer properties (Cotts *et al.*, 2000; Ittel *et al.*, 2000).

In the solid state, the structure exhibits *trans*-conformation about the central C—C bond of the ligand backbone. Bond lengths and angles are within the expected range for α-diimines. The dihedral angle between the aryl ring and 1,4-diazabutadiene plane is 78.23 (3)°(Fig. 1). In the crystal packing, there is no hydrogen-bond between the molecules.

### Experimental

Formic acid (0.5 ml) was added to a stirred solution of 2,3-butanedione (0.042 g, 0.49 mmol) and 4-Bromo-2,6-diisopropyl-phenylamine (0.250 g, 0.98 mmol) in methanol (20 ml). The mixture was refluxed for 24 h, then cooled and the precipitate was separated by filtration. The solid was recrystallized from dichloromethane/cyclohexane (*v*/*v* = 8:1), washed with cold ethanol and dried under vacuum to give the title ligand 0.21 g (75%). Anal. Calcd. for C_28_H_38_Br_2_N_2_: C, 59.79; H, 6.81; N,4.98; Found: C, 60.29; H, 6.95; N, 4.74.

### Refinement

All hydrogen atoms were placed in calculated positions with C—H distances of 0.93 and 0.96 Å for aryl and methyl type H-atoms. They were included in the refinement in a riding model approximation, respectively. The H-atoms were assigned *U*iso = 1.2 times *U*eq of the aryl C atoms and 1.5 times *U*eq of the methyl C atoms.

### Crystal data

**Table:**

C_28_H_38_Br_2_N_2_	*F*(000) = 580
*M**_r_* = 562.42	*D*_x_ = 1.284 Mg m^−^^3^
Monoclinic, *P*2_1_/*n*	Mo *K*α radiation, λ = 0.71073 Å
Hall symbol: -P 2yn	Cell parameters from 1370 reflections
*a* = 9.099 (3) Å	θ = 2.9–21.4°
*b* = 12.199 (4) Å	µ = 2.80 mm^−^^1^
*c* = 13.566 (5) Å	*T* = 296 K
β = 104.905 (5)°	Block, yellow
*V* = 1455.2 (9) Å^3^	0.25 × 0.23 × 0.19 mm
*Z* = 2	

### Data collection

**Table:**

Bruker APEXII CCD diffractometer	2685 independent reflections
Radiation source: fine-focus sealed tube	1460 reflections with *I* > 2σ(*I*)
Graphite monochromator	*R*_int_ = 0.043
φ and ω scans	θ_max_ = 25.5°, θ_min_ = 2.3°
Absorption correction: multi-scan (*SADABS*; Bruker, 2001)	*h* = −10→10
*T*_min_ = 0.541, *T*_max_ = 0.618	*k* = −12→14
7266 measured reflections	*l* = −16→14

### Refinement

**Table:**

Refinement on *F*^2^	Primary atom site location: structure-invariant direct methods
Least-squares matrix: full	Secondary atom site location: difference Fourier map
*R*[*F*^2^ > 2σ(*F*^2^)] = 0.052	Hydrogen site location: inferred from neighbouring sites
*wR*(*F*^2^) = 0.179	H-atom parameters constrained
*S* = 0.94	*w* = 1/[σ^2^(*F*_o_^2^) + (0.1*P*)^2^ + 0.7029*P*] where *P* = (*F*_o_^2^ + 2*F*_c_^2^)/3
2685 reflections	(Δ/σ)_max_ < 0.001
150 parameters	Δρ_max_ = 0.46 e Å^−^^3^
84 restraints	Δρ_min_ = −0.40 e Å^−^^3^

### Special details

**Table:**

Geometry. All e.s.d.'s (except the e.s.d. in the dihedral angle between two l.s. planes) are estimated using the full covariance matrix. The cell e.s.d.'s are taken into account individually in the estimation of e.s.d.'s in distances, angles and torsion angles; correlations between e.s.d.'s in cell parameters are only used when they are defined by crystal symmetry. An approximate (isotropic) treatment of cell e.s.d.'s is used for estimating e.s.d.'s involving l.s. planes.
Refinement. Refinement of *F*^2^ against ALL reflections. The weighted *R*-factor *wR* and goodness of fit *S* are based on *F*^2^, conventional *R*-factors *R* are based on *F*, with *F* set to zero for negative *F*^2^. The threshold expression of *F*^2^ > σ(*F*^2^) is used only for calculating *R*-factors(gt) *etc*. and is not relevant to the choice of reflections for refinement. *R*-factors based on *F*^2^ are statistically about twice as large as those based on *F*, and *R*- factors based on ALL data will be even larger.

### Fractional atomic coordinates and isotropic or equivalent isotropic displacement parameters (Å^2^)

**Table:**

	*x*	*y*	*z*	*U*_iso_*/*U*_eq_	
Br1	0.35688 (8)	0.65673 (6)	0.10127 (7)	0.0922 (4)	
C1	0.7029 (6)	0.8851 (4)	0.1392 (4)	0.0489 (12)	
C2	0.5759 (6)	0.8270 (4)	0.1476 (4)	0.0533 (14)	
H2	0.5247	0.8477	0.1958	0.064*	
C3	0.5254 (6)	0.7392 (5)	0.0851 (4)	0.0539 (14)	
C4	0.5971 (7)	0.7079 (4)	0.0117 (4)	0.0557 (15)	
H4	0.5608	0.6482	−0.0301	0.067*	
C5	0.7232 (7)	0.7651 (4)	−0.0001 (5)	0.0585 (14)	
C6	0.7756 (6)	0.8543 (4)	0.0646 (4)	0.0452 (13)	
C7	0.7611 (8)	0.9814 (5)	0.2083 (5)	0.0662 (14)	
H7	0.8701	0.9872	0.2130	0.079*	
C8	0.6907 (11)	1.0865 (6)	0.1624 (7)	0.126 (3)	
H8A	0.7162	1.0991	0.0990	0.188*	
H8B	0.7283	1.1459	0.2084	0.188*	
H8C	0.5822	1.0819	0.1505	0.188*	
C9	0.7469 (11)	0.9636 (8)	0.3149 (6)	0.120 (3)	
H9A	0.8150	1.0122	0.3607	0.180*	
H9B	0.7725	0.8891	0.3348	0.180*	
H9C	0.6443	0.9783	0.3175	0.180*	
C10	0.8059 (9)	0.7265 (6)	−0.0773 (6)	0.0804 (16)	
H10	0.8666	0.7876	−0.0923	0.096*	
C11	0.9126 (12)	0.6347 (7)	−0.0315 (8)	0.132 (3)	
H11A	0.9847	0.6606	0.0287	0.199*	
H11B	0.9656	0.6101	−0.0802	0.199*	
H11C	0.8555	0.5750	−0.0139	0.199*	
C12	0.6986 (12)	0.6892 (8)	−0.1768 (7)	0.126 (3)	
H12A	0.6336	0.6323	−0.1632	0.189*	
H12B	0.7566	0.6616	−0.2214	0.189*	
H12C	0.6378	0.7500	−0.2086	0.189*	
C13	0.9228 (6)	0.9765 (4)	−0.0034 (4)	0.0512 (14)	
C14	0.7932 (7)	1.0159 (6)	−0.0879 (5)	0.081 (2)	
H14A	0.8104	0.9956	−0.1523	0.122*	
H14B	0.7856	1.0942	−0.0843	0.122*	
H14C	0.7003	0.9831	−0.0812	0.122*	
N1	0.9141 (5)	0.9060 (3)	0.0633 (3)	0.0504 (11)	

### Atomic displacement parameters (Å^2^)

**Table:**

	*U*^11^	*U*^22^	*U*^33^	*U*^12^	*U*^13^	*U*^23^
Br1	0.0812 (6)	0.0846 (6)	0.1286 (8)	−0.0381 (4)	0.0595 (5)	−0.0188 (4)
C1	0.053 (3)	0.045 (3)	0.054 (3)	−0.005 (2)	0.023 (2)	−0.001 (2)
C2	0.053 (3)	0.054 (3)	0.058 (4)	−0.001 (3)	0.023 (3)	−0.001 (3)
C3	0.055 (3)	0.052 (3)	0.060 (4)	−0.011 (3)	0.026 (3)	0.005 (3)
C4	0.070 (4)	0.034 (3)	0.066 (4)	−0.014 (3)	0.024 (3)	−0.004 (3)
C5	0.075 (3)	0.043 (3)	0.070 (3)	−0.012 (3)	0.043 (3)	0.001 (3)
C6	0.046 (3)	0.037 (3)	0.055 (3)	−0.005 (2)	0.017 (3)	0.008 (2)
C7	0.076 (3)	0.062 (3)	0.067 (3)	−0.015 (3)	0.028 (3)	−0.015 (3)
C8	0.145 (7)	0.070 (5)	0.145 (6)	−0.001 (5)	0.005 (6)	−0.036 (5)
C9	0.155 (7)	0.131 (6)	0.083 (5)	−0.052 (5)	0.045 (5)	−0.038 (5)
C10	0.100 (4)	0.066 (3)	0.095 (4)	−0.016 (3)	0.060 (3)	−0.018 (3)
C11	0.131 (7)	0.129 (6)	0.163 (7)	0.022 (5)	0.085 (6)	−0.026 (6)
C12	0.153 (7)	0.147 (6)	0.100 (6)	−0.030 (6)	0.073 (5)	−0.040 (5)
C13	0.053 (3)	0.045 (3)	0.059 (4)	−0.006 (2)	0.019 (3)	0.007 (3)
C14	0.059 (4)	0.083 (5)	0.093 (5)	−0.012 (3)	0.005 (4)	0.039 (4)
N1	0.052 (3)	0.044 (3)	0.059 (3)	−0.006 (2)	0.022 (2)	0.005 (2)

### Geometric parameters (Å, º)

**Table:**

Br1—C3	1.894 (5)	C9—H9A	0.9600
C1—C2	1.384 (7)	C9—H9B	0.9600
C1—C6	1.396 (7)	C9—H9C	0.9600
C1—C7	1.511 (8)	C10—C11	1.507 (11)
C2—C3	1.371 (7)	C10—C12	1.517 (11)
C2—H2	0.9300	C10—H10	0.9800
C3—C4	1.376 (8)	C11—H11A	0.9600
C4—C5	1.387 (7)	C11—H11B	0.9600
C4—H4	0.9300	C11—H11C	0.9600
C5—C6	1.402 (7)	C12—H12A	0.9600
C5—C10	1.513 (8)	C12—H12B	0.9600
C6—N1	1.414 (7)	C12—H12C	0.9600
C7—C8	1.495 (10)	C13—N1	1.265 (6)
C7—C9	1.501 (9)	C13—C14	1.497 (8)
C7—H7	0.9800	C13—C13^i^	1.498 (10)
C8—H8A	0.9600	C14—H14A	0.9600
C8—H8B	0.9600	C14—H14B	0.9600
C8—H8C	0.9600	C14—H14C	0.9600
			
C2—C1—C6	118.8 (5)	H9A—C9—H9B	109.5
C2—C1—C7	120.9 (5)	C7—C9—H9C	109.5
C6—C1—C7	120.2 (5)	H9A—C9—H9C	109.5
C3—C2—C1	120.2 (5)	H9B—C9—H9C	109.5
C3—C2—H2	119.9	C11—C10—C5	109.2 (6)
C1—C2—H2	119.9	C11—C10—C12	110.1 (7)
C2—C3—C4	121.2 (5)	C5—C10—C12	112.8 (6)
C2—C3—Br1	119.7 (4)	C11—C10—H10	108.2
C4—C3—Br1	119.0 (4)	C5—C10—H10	108.2
C3—C4—C5	120.3 (5)	C12—C10—H10	108.2
C3—C4—H4	119.8	C10—C11—H11A	109.5
C5—C4—H4	119.8	C10—C11—H11B	109.5
C4—C5—C6	118.4 (5)	H11A—C11—H11B	109.5
C4—C5—C10	119.8 (5)	C10—C11—H11C	109.5
C6—C5—C10	121.7 (5)	H11A—C11—H11C	109.5
C1—C6—C5	121.1 (5)	H11B—C11—H11C	109.5
C1—C6—N1	118.6 (5)	C10—C12—H12A	109.5
C5—C6—N1	119.9 (5)	C10—C12—H12B	109.5
C8—C7—C9	113.0 (7)	H12A—C12—H12B	109.5
C8—C7—C1	111.3 (5)	C10—C12—H12C	109.5
C9—C7—C1	112.5 (5)	H12A—C12—H12C	109.5
C8—C7—H7	106.5	H12B—C12—H12C	109.5
C9—C7—H7	106.5	N1—C13—C14	125.7 (5)
C1—C7—H7	106.5	N1—C13—C13^i^	116.6 (6)
C7—C8—H8A	109.5	C14—C13—C13^i^	117.8 (6)
C7—C8—H8B	109.5	C13—C14—H14A	109.5
H8A—C8—H8B	109.5	C13—C14—H14B	109.5
C7—C8—H8C	109.5	H14A—C14—H14B	109.5
H8A—C8—H8C	109.5	C13—C14—H14C	109.5
H8B—C8—H8C	109.5	H14A—C14—H14C	109.5
C7—C9—H9A	109.5	H14B—C14—H14C	109.5
C7—C9—H9B	109.5	C13—N1—C6	122.2 (4)
			
C6—C1—C2—C3	1.6 (8)	C4—C5—C6—N1	172.3 (5)
C7—C1—C2—C3	−179.3 (5)	C10—C5—C6—N1	−4.1 (9)
C1—C2—C3—C4	−1.3 (9)	C2—C1—C7—C8	−90.6 (8)
C1—C2—C3—Br1	177.3 (4)	C6—C1—C7—C8	88.5 (8)
C2—C3—C4—C5	0.2 (9)	C2—C1—C7—C9	37.4 (8)
Br1—C3—C4—C5	−178.4 (4)	C6—C1—C7—C9	−143.5 (6)
C3—C4—C5—C6	0.4 (9)	C4—C5—C10—C11	−81.6 (8)
C3—C4—C5—C10	176.9 (6)	C6—C5—C10—C11	94.7 (8)
C2—C1—C6—C5	−0.9 (8)	C4—C5—C10—C12	41.1 (9)
C7—C1—C6—C5	180.0 (5)	C6—C5—C10—C12	−142.6 (7)
C2—C1—C6—N1	−173.4 (5)	C14—C13—N1—C6	0.5 (9)
C7—C1—C6—N1	7.5 (8)	C13^i^—C13—N1—C6	−179.2 (6)
C4—C5—C6—C1	−0.1 (8)	C1—C6—N1—C13	−106.2 (6)
C10—C5—C6—C1	−176.4 (6)	C5—C6—N1—C13	81.2 (7)

Symmetry code: (i) −*x*+2, −*y*+2, −*z*.
